# Management of the slowly emerging zoonosis, Hendra virus, by private veterinarians in Queensland, Australia: a qualitative study

**DOI:** 10.1186/s12917-014-0215-6

**Published:** 2014-09-17

**Authors:** Diana H Mendez, Jenny Kelly, Petra Buttner, Madeleine Nowak, Rick Speare

**Affiliations:** College of Public Health, Medical and Veterinary Sciences, James Cook University, Townsville, QLD 4811 Australia; College of Medicine and Dentistry, James Cook University, Townsville, QLD 4811 Australia; Centre for Nursing and Midwifery Research, James Cook University, Townsville, QLD 4811 Australia; Tropical Health Solutions Pty Ltd, Townsville, Queensland 4811 Australia

**Keywords:** Veterinarians, Emerging infectious disease, Zoonoses, Hendra virus, Infection control, Management, Behavioural change, Queensland

## Abstract

**Background:**

Veterinary infection control for the management of Hendra virus (HeV), an emerging zoonosis in Australia, remained suboptimal until 2010 despite 71.4% (5/7) of humans infected with HeV being veterinary personnel or assisting a veterinarian, three of whom died before 2009. The aim of this study was to identify the perceived barriers to veterinary infection control and HeV management in private veterinary practice in Queensland, where the majority of HeV outbreaks have occurred in Australia.

**Results:**

Most participants agreed that a number of key factors had contributed to the slow uptake of adequate infection control measures for the management of HeV amongst private veterinarians: a work culture characterised by suboptimal infection control standards and misconceptions about zoonotic risks; a lack of leadership and support from government authorities; the difficulties of managing biosecurity and public health issues from a private workforce perspective; and the slow pattern of emergence of HeV. By 2010, some infection control and HeV management changes had been implemented. Participants interviewed agreed that further improvements remained necessary; but also cautioned that this was a complex process which would require time.

**Conclusion:**

Private veterinarians and government authorities prior to 2009 were unprepared to handle new slowly emerging zoonoses, which may explain their mismanagement of HeV. Slowly emerging zoonoses may be of low public health significance but of high significance for specialised groups such as veterinarians. Private veterinarians, who are expected to fulfil an active biosecurity and public health role in the frontline management of such emerging zoonoses, need government agencies to better recognise their contribution, to consult with the veterinary profession when devising guidelines for the management of zoonoses and to provide them with greater leadership and support. We propose that specific infection control guidelines for the management of slowly emerging zoonoses in private veterinary settings need to be developed.

## Background

Hendra virus (HeV) is amongst a plethora of zoonoses emerging as new infectious threats to animal as well as human health [1]. HeV first emerged in 1994 in Brisbane, Queensland, Australia [2,3]. HeV is a paramyxovirus carried by pteropid bats with low infectivity to other susceptible species [4,5]. However, the virus can sporadically spillover from its natural reservoir to horses with secondary spillover to humans in some instances. HeV has high fatality rates in both species, 75% and 57% respectively [6]. Humans can become infected with HeV when exposed to blood and bodily fluids from an infected horse [2,3,7-11]. In 2011, a dog became infected with HeV but did not develop any clinical signs prior to being sacrificed [12]. Since 1994 there have been a relatively low number (49) of self-limiting outbreaks along the eastern coast of Australia between northern New South Wales and Far North Queensland [6]. These outbreaks resulted in the deaths of 90 horses and a total of three non-fatal and four fatal human infections [6]. So far, all human HeV cases have occurred between August 1994 and September 2009 in Queensland [6].

HeV infection in humans can be prevented by avoiding contact with infected horses or avoiding exposure to infected substances by implementing infection control (IC) measures such as using personal protective equipment (PPE) [13]. Administration of monoclonal antibodies during the incubation period has been shown to prevent experimentally induced HeV disease in ferrets and non-human primate models and this has also been used in humans exposed to HeV as an experimental prophylaxis [14]. A vaccine for horses became available in late 2012 and was promoted for the protection of horse populations from HeV and humans from secondary spillover [15,16].

Since the emergence of HeV the veterinary profession has paid the highest price with the death of two veterinarians, a veterinary assistant and the non-fatal infection of a veterinarian and a veterinary nurse [2,3,6-11]. As a result of these HeV-related deaths, Federal and State governments and professional agencies initiated an IC and HeV management campaign targeted at equine veterinary practices. Despite the early information campaigns about HeV related risks and risk mitigation strategies targeting private veterinarians, the last two human cases of HeV occurred as late as 2008 and 2009 and proved fatal for two veterinarians, one of whom had recently attended a HeV management workshop [6,9-11]. Additionally, an Australian study conducted in early 2010 by a State government agency showed that IC and HeV management practices in private equine veterinary clinics were still suboptimal particularly when carrying out high risk procedures (such as dental procedures, endoscopy of the upper respiratory or urinary tracts or endotracheal intubation) with high likelihood of exposure to oral, respiratory and urogenital bodily fluids from a healthy or a sick horse [17]. As a result, government authorities and veterinary professional agencies questioned why private veterinarians were still becoming infected with HeV. In mid to late 2010, in an attempt to promote changes in HeV management and related IC behaviours, the Queensland government sent a new comprehensive HeV management information package to all private veterinarians registered in Queensland (Dr B. Pott, personal communication).

The aim of this paper is to explore the barriers to IC and HeV management in private practices prior to September 2010 as perceived by equine veterinarians and allied staff in Queensland, Australia.

## Methods

### Research protocol

The purpose of this qualitative study was to identify and understand the factors affecting IC and HeV management in veterinary private practices in Queensland, Australia. This study was conducted as an exploratory, descriptive design using semi-structured interviews. This method was chosen in order to gain an in-depth understanding of the perceptions and experiences related to the management of HeV and IC implementation in private practices in Queensland [18-20]. No adequate research tool had been previously developed to conduct such a study in the context of HeV emergence. After consultation with major stakeholders involved in the management of HeV (private equine veterinary practitioners, lecturers in equine medicine and representatives from professional and State government agencies) a series of open-ended interview questions were formulated to explore HeV related risk perceptions as well as barriers to IC and HeV management in equine veterinary practices in Queensland. The interview questions were piloted with one equine veterinarian and questions modified accordingly.

Between December 2009 and September 2010 individual, face-to-face interviews were conducted with 21 veterinary personnel from 14 veterinary practices in rural and urban areas along the eastern coast of Queensland, Australia. Interviews were semi-structured to allow the in-depth exploration of issues relating to barriers to IC and HeV management in the context of their veterinary work as perceived by the participating veterinary staff [20]. This project was conducted with the approval of the James Cook University Human Ethics Committee (permit H3513) and complied with qualitative research guidelines on relevance, appropriateness, transparency and soundness of research methodology (RATS) [21,22].

### Recruitment and interview protocols

Participation was voluntary and participants could withdraw from the study at any time. Prospective suitable veterinary practices were identified from public phone directories and their associated advertisements and web links. Potential participants were initially contacted by phone, by the lead interviewer (DM), at which time the purpose and protocol of the study were explained to them by the researcher. Prospective participants were subsequently provided via e-mail with a project information sheet and explanations about what would be required of them at the time of the interview. Some declined to participate without further explanation while others were “too busy” or suspicious of the research process. Upon agreement to participate, a suitable interview date, time and location for each participant were organised. At the time of the interviews, the lead interviewer (DM) provided further information about the project and the study protocol when requested by a participant prior to the signing of the consent form. In particular, many participants were concerned about retaining anonymity and wished to ascertain their responses would remain unidentified. This was assured. Prior to commencing each interview, participants were required to provide socio-demographic and professional profile information such as the type of practice they worked in, their role in the practice, an estimation of percentage of their working time spent providing veterinary services to horses and their experience with HeV outbreaks. Recruitment of new participants ceased when data saturation was reached; i.e., interviews stopped yielding new perspectives. This occurred after interview number 18.

### Participants

The target population were veterinary personnel working in private practice in Queensland. The eligibility criteria were: 1) working in private veterinary practice providing veterinary services to either horses exclusively (i.e., equine practice) or to horses as well as to other large and small domestic animals (i.e., mixed practice); 2) working within the known geographic distribution area of HeV in Queensland: the eastern coast between the Far North and the Queensland-New South Wales border [23]. In order to collect a comprehensive range of views within the target population, participants, with different socio-demographic and professional profiles, were purposively selected from regions where HeV outbreaks had occurred (South East; Central; North; and Far North of Queensland). The final study population comprised 21 participants from a total of 14 mixed private veterinary practices. According to the Australia Rural, Remote and Metropolitan Areas (RRMA) classification system nine participants were from areas classified as metropolitan, nine were from rural areas and three were from remote areas [24]. The majority of participants (18) were veterinarians; two were experienced female veterinary nurses and one was a male practice manager who although having no direct involvement with equine patients, worked in a practice with a strong equine component. In most cases, the first point of contact during the recruitment process was the principal veterinarian owner of the practice. More than half the veterinarians interviewed were principal veterinarians (10) of whom only one was female. Interviews with principal veterinarians lasted, on average, longer (75.6 minutes) than interviews with other participants (46 minutes). The remaining eight veterinarians were partners/associates or employees, five of whom were female. Females were on average younger and aged between 31 and 48 years old while males were aged between 28 and 63 years old. This was reflected in the difference of the average number of years since graduation between males (mean = 26.4 yrs, range: 4–40 yrs) and females (mean = 13.1 yrs; range: 4–27 yrs). Excluding the practice manager, on average, females spent proportionally less of their working time than males attending to horse patients (30.4% vs 52.1%). Prior to this study there had only been 13 confirmed outbreaks of HeV with one outbreak occurring during the data collection period [6]. However, more than half of the participants (60%) had dealt with at least one suspected case of equine HeV and over a third (35%) had dealt with at least one confirmed equine HeV case prior to being interviewed. Further details of this study population have been described elsewhere [25-27].

### Data collection

For convenience, the majority of interviewees chose to be interviewed in an office at their place of work or in their own homes. The interviews were conducted by the lead author (DM), a female veterinarian with research skills in qualitative interview techniques, who was well aware of the issues under investigation, with a research assistant participating in some interviews. With written consent from each participant, responses were recorded as digital audio files with complementary hand written notes. Participants were asked to share their experiences when dealing with HeV and what, in their opinion, were the barriers to IC and WHS compliance. At the conclusion of each interview, the notes were read back to each participant to give them the opportunity to clarify or amend their responses. Audio files were later transcribed and entered into qualitative data analysis software (NVivo qualitative data analysis software; QSR International Pty Ltd. Version 9, 2010). After each interview, the interviewer reviewed the notes and recorded a number of reflections capturing the context of each interview. Interviews took between 40 and 160 minutes, with an average time of 64 minutes.

### Data analysis

Prior to analysis, each participant was identified using a unique alphanumeric code (V* for veterinarians, VN* for veterinary nurses and PM* for practice manager) and a unique alphanumeric veterinary practice code (P*). Thematic analysis was used to analyse the qualitative data [28]. Interview transcripts were analysed with NVivo (QSR International Pty Ltd. Version 9, 2010) for repeating units of meaning and the main themes and subthemes were conceptualised using inductive thematic analysis [20,28]. This process was repeated on more than one occasion with identified themes discussed and agreed upon by three of the researchers (DM, RS, JK). Two of the researchers were veterinarians and thus were able to review the themes within the context of private veterinary practice and the emergence of HeV and its management.

## Results

Data analysis revealed six main themes expressing the participants’ responses about the difficulties associated with the implementation of adequate IC and HeV management in private veterinary practices between the first outbreak in 1994 and the time of the interviews (2009–2010) (Table 1).These themes are: the emerging epidemiology of HeV; risk perception determines risk mitigation; risk and risk mitigation communication; education and work culture; personal protective equipment; and running a private veterinary practice.

**Table 1:** **Main themes surrounding the topics of barriers to infection control and Hendra virus management in private veterinary practices identified in Queensland, Australia, between 2009 and 2010, as illustrated by participants’ quotes**

**Themes**	***Examples of quotes from participants’ responses which best illustrate the themes***
**The emerging epidemiology of the virus**	“*When the first case of Hendra came about, it was seen as being rare so we didn’t see* [it] as a *problem.*” (V3/Pa)
	“*It would have been better to have known more about the virus. Where do you draw the line when you don’t know enough about the risks and the non-risks?*” (V4/Pc)
	“*The drive* [for IC*] *needs to be continuous…HeV cases are seldom, so it’s easy for people to be slack, to back off from infection control*.” (V10/Pg)
	“*The biggest challenge is how to manage a hospital facility for horses, because the case definition is not clear…Our biggest concern…a horse that shows up without Hendra on the differential list and it* [HeV^#^] *shows up on the list later*.” (V16/Pl)
	“*This is a disease that is here to stay….so we have to learn to live with it.*” (V15/Pk)
**Risk perception determines risk mitigation**	“*Some think that Hendra is only a North Queensland problem and that it wouldn’t happen in South Queensland or northern New South Wales.”* (V7/Pa)
	“*I didn’t think a great deal about it* [HeV] *because I studied in Victoria and it was presented to us as a disease only found in Queensland*.” (V14/Pj)
	*“No way in the world would I put a mask or gloves on unless I saw something dramatic.”*(V13/Pi)
	*“You will not get a primary Hendra case with a horse living in a stable.”* (V1/Pa)
	*“At the time* [before HeV]*, human safety was not being kicked* [by a horse patient], *not being trampled on, avoiding any physical injury…Zoonoses were not a big one on my list.”* (V14/Pj)
	“*Seeing a mate die I think is enough. Obviously you think of self-preservation. I have a life to live…Dying is a pretty big cost.*” (V5/Pd)
**Risk and risk mitigation communication**	*“There were no guidelines in place in regards to what* [protective] *equipment should be worn…there was information on what samples were needed but not how to take them.”* (V14/Pj)
	*“The trouble with AVA* ^*†*^ *is that there is only 50% membership…weekends are when the AVA has the workshops and that is when we have a lot of work on here.”* (V15/Pk)
	*“Most vets would receive information through email. Some think it’s not enough but some of those don’t read their emails*.” (V1/Pa)
	*“They* [people at the DPI^‡^] *are excellent. The DPI is the most authoritative source of knowledge because they have been dealing with the issues and they are responsible for biosecurity.”* (V15/Pk)
	*“The local DPI were useless, almost burying their heads in the sand. They didn’t have enough knowledge, were unwilling to investigate and had a lack of resources and people.”* (V9/Pf)
	*“I contacted Biosecurity Queensland on a Saturday afternoon…They transferred me on the phone 19 times to talk to a vet… they have to realise that private vets work 24/7…This disease is too serious to be ignored…they should have been more responsive. It would have been easier to put the horse in a hole and forget about it!”* (V5/Pd)
	*“I find great difficulty dealing with owners because it is a power play and ultimately we are responsible for the safety of all involved but some owners don’t believe that, which compromises the legal situation. We usually end up with less authority out of concern for the welfare of the animal.”* (V4/Pc)
	*“By the time you get to the horse you are only the 3rd or 4th opinion…A lot of people around here are gung ho who*[think they] *know better.”* (V5/Pd)
	*“Some owners burr at the cost…He* [the owner] *said ‘if you want to test the horse why don’t you pay for it’.*” (V14/Pj)
	“[The owner said] *I don’t want them tested for Hendra. If they turn up positive they’ll have to be put down. My horses are like my children and I wouldn’t euthanase my own children.*” (V9/Pf)
**Education and work culture**	“*Students are very well aware of the risks to the point of being scared to do things.*” (V7/Pa)
	“*These days young vets lack confidence around horses because of their lack of experience through their training and personal life.*” (V1/Pa)
	*“Coming out of Uni the biggest issue was not whether I could follow protocols it was getting the practice of using those protocols.”* (V17/Pm)
	“[I]*had been inadequately trained* [about HeV] *when studying in Victoria* [another state] *…I didn’t realise that interstate* [studies] *did not offer the same information. Some things should not be disregarded in the curriculum because it doesn’t happen in that area.*” (V14/Pj)
	*“Hopefully they* [the students] *will go into practice in clinics where they follow best practice.”* (V7/Pa)
	*“Poor mentorship is a problem. Training is very important. A principal vet can teach a lot to younger vets.”* (V8/Pe)
	“*I think overall we, as vets, were pretty grubby.*..*to get around with blood on your shirt all day that is just what veterinarians did.*” (V16/Pl)
	*“Old school vets always considered getting infected with a zoonosis as a badge of honour.”* (V8/Pe)
	*“The biggest obstacle is trying to retrain someone who has done something a certain way for 30 odd years.”* (V13/Pi)
	*“The culture needs to change*…[but]…*change won’t happen overnight. Human medicine was hit in the 1980s* [by HIV^Ω^], *it sparked infection control improvement but it’s still not perfect now. The* [veterinary] *profession has to change but this will take time.”* (V8/Pe)
**Use of Personal Protective Equipment**	*“If I am going to be completely honest, a few years ago there would be no way I’d put it* [PPE^◊^] *on.”* (V13/Pi)
	“*We have always been casual about horses. In cattle you have brucellosis but I have never been worried about catching anything from horses.”* (3/Pa)
	*“The right PPE … was not available at the clinic at the time. PPE was still not considered to be an issue at the time.”* (V10/Pg)
	“*You can read the protocol all you like but until you actually do it and take the gear off under the assumption that you are contaminated it is not an easy thing to do.”* (V12/Ph)
	“*By following the DPI protocol it took us two hours before the animal was finally sedated.”* (V4/Pc)
	*“PPE can be cumbersome…smelling the breath of a horse gives you an idea of what’s going on. If you are wearing a mask it makes it difficult.”* (V11/Pg)
	*“The ability to move properly…can be a problem. When you have to deal with horses with fractures you have to move quickly!”* (V10/Pg)
	*“The biggest obstacle: heat and humidity…When you are wearing the full PPE it can be very hot.*” (V6/Pa)
	*“I hate masks, they press on the bottom of my eyes and I can’t see…I won’t wear goggles it’s too difficult with my glasses.”* (V5/Pd)
	*“Comfort and practicability is an issue for me because one size fits none!!…PPE suits are baggy on me, with a lot of flapping about and this scares horses.”* (V7/Pa)
	*“During the EI* ^*§*^ *campaign I had to vaccinate between 800 and 1000 horses and at each farm I had to put on a new set of PPE so it’s not a big issue for me.” *(V2/Pb)
	*“Cost is a big one. You have to pass the cost on. The bill can be a lot dearer than before and if the horse turns out negative, the owner will whinge about the money spent.”* (V5/Pd)
	*“We are lucky in that our clients are used to spending money on their horses.”* (V12/Ph)
	*“It is potentially dangerous…to have two people walk up* [to a horse] *looking like spacemen with their clothes rustling and their voices distorted.”* (V12/Ph)
	*“Vets like me don’t want to be seen with over the top PPE…in more rural situations you tend to think you are being a bit of a Wally*[silly] *dressed up for minor issues.*” (V10/Pg)
	*“…You felt silly in front of other colleagues to be wearing all the gear.”* (V3/Pa)
	“*Clients could be a driving force behind it* [IC] *because they would start questioning why some vets use PPE and others don’t.”* (V1/Pa)
**Running a private veterinary practice**	*“When you have a big backlog of work there is time pressure… your logistics are stretched…many cases end up being emergency cases at the end of the day, which is a recipe for disaster because you start cutting corners and making mistakes.”* (V4/Pc)
	*“Vets are often busy and to get through a large case load is hard enough, let alone stopping to put protective gear on.”* (V16/Pl)
	*“Cost is an issue …Do you then transfer the cost onto the owner? What if the case turns out negative? How do you justify it?”* (VN2/Pe)
	*“There would be cases out there that have not been reported because of the cost…I think this puts people at risk. As long as it is hurting the pocket of the clients or the pocket of the vet, cases will go unreported.”* (V14/Pj)
	*“In the hospital system everything is …disposable. If we had to treat everything as disposable our cost would increase…The industry would suffer from it.”* (V3/Pa)
	*“As vets we don’t have the luxury of the health system behind us to make* [IC] *decisions, we don’t have a large buying power… for vets the one size fits all approach doesn’t work”.* (V8/Pg)
	*“You look at the paper trail that they* [WHS^¥^ authorities] *say you need to have…you would have to employ somebody full time for six months to put it in place.”* (V12/Ph)
	*“As an employer you can do everything in your power and you can make all the recommendations but it comes down to the individual and if the individual doesn’t have Hendra on their mind there is not much you can do.”* (V14/Pj)
	*“Legally the only way you could send a staff to a property is if on the day the employer went to that property and addressed the risks and said it was safe to work there, because the conditions from yesterday might not apply today.”* (V9/Pf)
	*“More positive cases are going to land in our hospitals…Infection control is imperative but what scares me is the unknown* [when HeV is not readily suspected]*.”* (V16/Pl)
	*“The DPI* ^*‡*^ *contacted me to do this work and they weren’t being flexible about it: they wanted me there and then. This was [8 months ago] …I am still waiting for my money! Next they'd have to pay me up front.”* (V5/Pd)
	“*Everyone’s fear is you’ll act to the best of your ability and you’ll act in the best interest of everyone involved but later your actions will be audited by someone who has never worked in a vet practice and has no idea of the pressures at play and who will apply the letter of the law and potentially cost you your livelihood.”* (V12/Ph)
	*“I don’t talk to local* [WHS] *officers anymore because they don’t have enough knowledge. This immediately established a low level of credentials with us. Everything after that we see it more as an inconvenience.”* (V8/Pe)

*IC: Infection control; ^#^HeV: Hendra virus; ^†^AVA: Australian Veterinary Association; ^‡^DPI: Department of Primary Industries; ^Ω^HIV: Human Immunodeficiency Virus; ^◊^PPE: Personal protective equipment; ^§^EI: Equine influenza; ^¥^WHS: Workplace health and safety.

Each theme included a number of subthemes that are illustrated in Table 1 by selected quotes from participants’ responses. The quotes included in Table 1 were selected as best illustrations of the themes and subthemes identified in this study.

### The emerging epidemiology of the virus

Many participants were initially not overly concerned about HeV; the sporadic occurrence of equine cases and the rarity of human cases separated by long periods of time belied the seriousness of the epidemic. When HeV first emerged, participants reported that they and others in the profession failed to recognise the significance of this new zoonosis. Most participants felt that in the first few years of HeV emergence, there was insufficient knowledge about the disease and its epidemiology to adequately assess and therefore manage the risks involved. Nevertheless, when HeV began recurring more regularly, participants started implementing HeV management strategies, although these strategies were not always sustained over time. Another major stumbling block identified by many participants was the difficulty associated with the clinical diagnosis of the disease in horses. As some participants pointed out, the case-definition of HeV is non-specific and there can be substantial variations in clinical presentations which rendered the initiation of a HeV management plan difficult. Furthermore, an asymptomatic horse did not always equate to a healthy uninfected horse as it could be in the late incubation period of the disease during which viral shedding is possible. In 2010, however, most participants agreed that HeV was likely to recur in the future and that they needed to adopt a sustainable HeV management plan.

### Risk perception determines risk mitigation

Participants who failed to mitigate the HeV-related risks often perceived the risk of being exposed to HeV to be low. Their risk perceptions were initially based on geographic consideration. This viewpoint echoed that of other participants who recalled that prior to 2010 there was an ongoing belief that occurrence of HeV would be restricted to specific geographic areas outside of which the likelihood of HeV spilling over was low as was the risk of exposure. This belief seemed to be perpetuated outside the initial affected state of Queensland. Participants who had studied or started their career in other states were not overly concerned about HeV.

Hendra virus was generally perceived to be a more significant risk by participants if they initially included it in their differential diagnosis, which in turn determined their HeV management attitudes. However, many participants reported that prior to the last fatal human case in 2009, they did not perceive the HeV-related risk to be high unless the symptomatology of a horse was severe and that they would only have considered taking precautions in extreme clinical cases. Some participants were confident they could rule out HeV, and the related risks, on clinical examination alone despite HeV having a non-specific case definition. Another common belief amongst participants was that HeV could only spillover into horses kept in paddocks and therefore, examining a horse kept in a stable represented a low HeV risk.

Participants also prioritised risk management according to risk likelihood. Because of their experience prior to the emergence of HeV, many participants perceived the risk of contracting an infectious disease in equine practice significantly lower than sustaining a serious injury from a horse. Conversely, the HeV-related infectious risk appeared to be of greater relevance for participants who had had direct dealings with early HeV positive cases or personally knew one of the people who had been infected with HeV. For example, one participant, who had dealings with one of the early outbreaks, became more circumspect when dealing with sick horses; while another participant was reconsidering the viability of managing equine cases after losing a close colleague to HeV.

### Risk and risk mitigation communication

At the time this study was conducted, participants were seeking and receiving information about the HeV from government and professional agencies such as the department of primary industries (DPI)/Biosecurity Queensland, the Australian Veterinary Association (AVA) and Equine Veterinarians Australia (EVA). However, not all information was perceived as complete, useful, specific or practical enough in assisting them with HeV management in the field. Additionally, not all information was accessible by all veterinarians. Some modes of information delivery, such as emails, were described as being ineffective in reaching the entirety of its target population; and professional associations could only reach their veterinary membership.

The majority of participants from or around large urban centres were mostly satisfied with the support they received from government agencies in charge of biosecurity. In contrast, participants from rural and remote areas were mostly dissatisfied with the level of support they received from the local representatives of the same government agencies. Government officers from these areas were perceived as lacking capacity, knowledge and experience.

Some participants from rural and remote areas further cautioned that the lack of responsiveness from the government at the local level could lead in some instances to potential cases of HeV going unreported and/or un-investigated.

Risk and risk mitigation communication also occurred between veterinary staff and horse owners. Some participants found risk communication with horse owners challenging as some clients were not receptive to veterinary directives about HeV-related risks. Many participants thought that the three main reasons for horse owners to refuse veterinary decisions were due to denial of the risk, cost issues and emotional attachment between owners and their animals.

### Education and work culture

Australian and overseas educated early career veterinarians who worked in the participating practices were mostly perceived by interviewees as being well informed about IC and HeV related risks. However, a number of participants thought that some undergraduate Australian veterinary students and newly graduated veterinarians lacked experience and confidence in applying IC and animal handling skills. Some participants recalled their own lack of confidence in applying IC principles earlier in their career which they thought was due to insufficient practical experience and variability in veterinary curricula between universities.

Other participants questioned the role of senior private veterinarians overseeing the practical training of veterinary students on extramural placements and early career veterinarians. Professional mentorship was reported as having a pivotal effect on veterinary IC training. In some instances, poor professional mentorship was deemed responsible for undergraduate students and early career veterinarians failing to adopt and develop adequate IC standards.

Participating senior veterinarians thought that their IC attitudes and beliefs about veterinary occupational risks, which were now considered suboptimal, were the norm at the beginning of their career. This enduring work culture was viewed as a major obstacle to IC improvement in veterinary practice as it required a drastic change in the way veterinarians thought, behaved and made decisions. However, most participants, regardless of how long they had worked in private practice, recognised that veterinary IC required amelioration but warned that it would take some time before this occurred.

### Use of personal protective equipment (PPE)

A number of participants, who began their career before the emergence of HeV, pointed out that prior to 1994 the use of PPE in equine practice was not a common occurrence. Some thought the lack of experience with zoonotic risk in equine practice led to the belief that the use of PPE for the management of HeV was not critical. By 2010 all participants were using some form of PPE in combination with other IC strategies to mitigate the risk of exposure to HeV and/or other zoonoses. However, some participants still found it difficult to use PPE routinely in equine practice. Participants gave a range of reasons why they thought the use of PPE presented some drawbacks: it sometimes hindered their ability to work efficiently, competently, safely and comfortably; or was deemed unsuitable for reasons of fit. Interestingly, participants who had repeatedly used PPE during the equine influenza outbreaks and vaccination campaigns did not perceive the use of PPE in equine practice to be an issue.

Some participants also thought that the systematic use of PPE had the disadvantage of increasing the running cost of a consultation which affected the veterinary practice as well as the client, although not all participants viewed the added PPE-related cost as a big issue.

A number of participants were also concerned about the way they were perceived by their patients, clients and colleagues when they wore PPE and the effect this would have on their workplace health and safety (WHS) and their professional credibility. Other participants thought that the clients who perceived the use of PPE as positive work practice could be a driving force behind equine veterinary practices more readily adopting the use PPE.

### Running a private veterinary practice

Time management was an issue for most veterinarians interviewed who reported that implementing additional IC measures affected work schedule, quality and safety. The extra time spent implementing additional IC measures was also reported to affect consultation fees. Many participants further explained that any additional cost, such as costs related to the management of HeV, needed to be justifiable to clients as it often affected their level of satisfaction with veterinary services provided. Cost was perceived by many participants as a limiting factor to the management of HeV from a client’s and a business perspective.

Some participants highlighted the discrepancies that existed between the business models of veterinary practices and public medical hospitals for people. They pointed out that veterinary practices were small businesses operating solely privately unlike medical hospitals and therefore could not be run as sustainably because of cost and logistical considerations.

Many participants were principal veterinarians who had the added responsibility of ensuring that the running of their practice, including the management of potential HeV cases, complied with the WHS legislation. However, many found that compliance was not easily achieved and in some instances hindered the running of the practice. The need to document WHS policies and procedures, training and incidents, for example, was perceived as unwelcomed extra work. The need to ensure the health and safety of staff who were not always receptive to warnings and instruction was viewed as another major challenge to compliance. Some participants thought that the legislation was open to interpretation; thus making it difficult to always comply. Many participants also felt that because compliance was not legally protective they remained vulnerable to unexpected situations. Although, most participants were aware that they could legally refuse to provide veterinary services if they deemed a situation unsafe, some were not afforded this option when being requested to fulfil HeV management duties on behalf of the government without receiving logistic support or compensation for their skilled services. Additionally, some participants thought that scrutiny of veterinary practices by WHS authorities was often inadequate and arbitrary to the detriment of their businesses.

## Discussion

Private veterinarians are likely to be the first frontline clinicians to encounter emerging zoonoses; which puts them at a higher occupational risk of exposure to these diseases [1,29]. Many studies, including Australian studies, have shown veterinarians’ attitudes and behaviours towards IC and zoonotic risks to be suboptimal; however, most studies have failed to explain why this was so [30-34]. The aim of this study was not to evaluate veterinary IC adequacy but to identify and understand the barriers to IC and zoonotic risk mitigation in private veterinary practices within the context of the emergence of HeV in Queensland, Australia. Participants reported experiencing difficulties implementing IC and HeV management for a number of reasons (Table 1). Many of these issues were interconnected and were in actual fact related to four main barriers to IC and HeV management discussed below: veterinary work culture; private workforce managing biosecurity and public health issues; role of government; and uncertainty about the epidemiology of an emerging disease (Table 2).

**Table 2:** **Summary of main barriers to infection control and Hendra virus management in private veterinary practices in Queensland, Australia, up until September 2010**

**Main issues**	***Related barriers***
**Work culture**	• *Longstanding observance of suboptimal IC practices;*
	• *Veterinarians’ perception that zoonotic risks in equine veterinary practice were low;*
	• *Veterinarians’ perception that they are more likely to be exposed to injury risks than infectious risks in equine practice;*
	• *Mitigation of injury risks more readily implemented by veterinarians than mitigation of infectious risks in equine practice;*
	• *Inadequate veterinary work habits perpetrated in some instances by poor professional mentorship during extramural undergraduate placement or during early career experiences.*
**Role of Government**	• *Suboptimal HeV testing pathways*
	• *Slow response from government authorities to the emergence of HeV and to HeV outbreaks*
	• *Suboptimal and conflicting communication of risk and risk mitigation from government authorities to veterinarians*
	• *Inconsistent government support for veterinarians throughout the state, with rural remote areas receiving less skilled technical support*
	• *Difficulties in complying and collaborating with WHS legislation and authorities*
**Managing animal and public health issues and a private business**	• *The logistical, financial and work time costs of implementing infection control changes within the context of running small private businesses*
	• *Difficulty in interpreting and enforcing WHS regulation*
	• *Mitigation of zoonotic risks interfering with the mitigation of injury risks*
	• *Lack of WHS legal protection when a third party breaches the legislation*
	• *Veterinarians’ lack of experience choosing and using some of the PPE recommended*
	• *Inadequate, insufficient and inconsistent training of undergraduate veterinarians about IC and HeV management*
	• *Difficulty in implementing IC behavioural changes amongst veterinary staff*
	• *Difficulty inefficiently communicating with clients about HeV-related risks and risk mitigation recommendations*
**Uncertainty about the epidemiology of an emerging disease**	• *Slow emergence and sporadic nature of HeV outbreaks*
	• *Slow gathering and dissemination of epidemiological information*
	• *Misinterpretation of epidemiological information*
	• *Non-specific HeV case definition*

### Strengths and limitations

Between December 2009 and September 2010, the issues surrounding veterinary IC and HeV management were sensitive topics amongst private veterinarians, as two of their colleagues had died of HeV in the previous two years [6,9-11]. Following these events private equine practices came under a high level of government scrutiny. As a result, prospective participants were reluctant to take part in the study. They were concerned their responses would be misunderstood, misrepresented and/or misused. Those who agreed to participate may have had “stronger views on” or “interest in” veterinary IC and HeV management because they had had experience with HeV or biosecurity and WHS government authorities; or because they were principal veterinarians, owners of their practice. Although data were collected over 10 months, the topics brought to the fore during the interviews were very similar and mostly related to veterinary IC and HeV management issues participants had experienced prior to 2010. Many of the views expressed by participants were corroborated by the findings of other reports about HeV management [35-37]. The chief investigator (RS) and the main interviewer (DM) are both veterinarians who were able to communicate with participants as colleagues who understood the context of their workplace. Consequently, participants were more open about their experiences and beliefs, which made for richer data. However, coding and thematic analysis were pursued without any preconceived construct other than the knowledge the researchers had of veterinary workplaces, allowing an in-depth understanding of the issues raised by participants.

### Veterinary work culture

Most participants agreed that, as a whole, the veterinary profession had initially been reluctant to adopt new IC strategies for the management of HeV because it required a significant shift in their work culture. Retrospectively, they felt unprepared to deal with an emerging zoonosis. They all agreed that when HeV first emerged veterinary IC was not optimal and needed improvement. In 2010 they were still trying to understand how to best manage HeV. A 2010 government study conducted within the same target population also concluded that IC for the management of HeV still needed amelioration [17]. However, participants cautioned this process would take time. By 2009–2010, all participants had made some improvements to their IC and HeV management strategies; some only recently while others had not been sustained long term. According to the hierarchy of control of health and safety risks which categorises risk mitigation strategies, the changes implemented varied greatly, with some participants implementing only low levels of control (using additional PPE); while others addressed IC issues at a much higher level of control (attending specialised training, developing new standard protocols and policies, seeking new engineered solutions) [38]. A few participants were still sceptical of the appropriateness of the recommended changes.

Successfully changing work culture in human healthcare settings has been described as a lengthy and complex process requiring strong leadership within an organisation [39,40]. The adoption of effective hand hygiene by healthcare workers is a good example. While hand hygiene was recognised in the late 1800s as the simplest and most effective IC measure that could help prevent healthcare associated infections, it remains a practice that is neither consistently nor adequately carried out by healthcare workers despite healthcare systems being strongly supported by government leadership [41]. In comparison, the push for veterinarians to adopt new HeV-related IC measures only dates from the last decade. Veterinarians communicated about HeV risk mitigation within and between practices via an informal professional network; however, most participants felt that government agencies in charge of biosecurity could have provided more leadership and support to the veterinary profession. Instead, the leadership in veterinary IC for the management of HeV was fragmented and vested in each principal veterinarian, the owner of his/her practice.

### Private workforce managing biosecurity and public health issues

Maintaining the financial viability and credentials of the veterinary practice, a small privately owned business, was a concern for all principal veterinarians and some senior veterinary employees interviewed. In their view any change in work habits/protocols could affect the business, presumably because of the perception that in private veterinary practice there is a cost ceiling and any additional cost would decrease profit. Profitability of the business is a common concern of veterinarians running private practices of all types [42,43]. The constraints of running a private business significantly influenced participants’ decisions about IC, and some found it difficult to balance their *de facto* public health and biosecurity roles while running a private business and complying with their WHS responsibilities. The WHS legislation, for example, was viewed as open to interpretation and difficult to implement in unpredictable circumstances not easily controlled. The difficulties of using some standard biosecurity measures in the field in equine practice are common to the management of other equine infectious diseases [44]. Some participants were aware that they could legally refuse provision of services if they deemed a situation unsafe [45]. However this option was considered as a last resort as it could jeopardise the welfare of animals, translate into loss of immediate income and future income if unsatisfied clients did not return to the practice. Refusing to provide veterinary services was not an option for participants who reported having been requested to fulfil HeV management duties on behalf of the government. For some, the weight of the legal responsibilities reached breaking point when HeV started to emerge, resulting in them choosing to exit equine veterinary practice [25]. So, although fear of dying acted as a significant motivator for some participants to implement lasting IC changes, the complexity of their responsibilities to their patients, clients, colleagues, business and the community deterred them from doing so.

### Role of the government

Many participants perceived government authorities’ slow response to the emergence of HeV as a sign of unpreparedness to deal with emerging zoonoses, which contributed to the slow uptake of new IC measures for the management of HeV management by private veterinarians. The subsequent increased scrutiny into veterinary practices by biosecurity and WHS government agencies was perceived by most participants as intrusive and an indication that they were regarded as the sole responsible for all HeV management shortcomings until 2010. However, most participants thought the government could have better contributed to the management of HeV outbreaks. An Ombudsman’s report about government response to HeV outbreaks concurred with this view [37]: prior to 2009 government agencies in charge of biosecurity failed to develop, finalise and implement their policies for the management of HeV and adequately train their staff accordingly. This was reported to have resulted in the communication of incomplete and conflicting information and poor field support of private veterinarians, despite previous independent reports identifying similar issues for the 2008 and 2009 outbreaks [35,36]. Investigation into the handling of equine influenza, an equine specific emerging disease in Australia in 2007, also showed the importance of risk communication from government to private veterinarians when managing emerging infectious diseases [46]. Many of the recommendations from the various reports about the management of HeV have since been adopted by the relevant government agencies [47]. Since 2010 no veterinary personnel have been infected, although equine cases have increased [6]. This may indicate that the provision of early leadership and committed support by government authorities to the veterinary profession may be key to implement more effective management plans of emerging zoonoses from both a biosecurity and a public health perspective.

### Uncertainty about the epidemiology of an emerging disease

The lack of preparedness was a major stumbling block in the early management of HeV by veterinarians and government authorities. The slow pattern of HeV emergence appears to have been a contributing factor in delaying the response of both the private and public sectors in charge of HeV management. The practice of IC for HeV management is a matter of biosecurity to protect animal health, of public health and of occupational health and safety. However, deciding to adopt adequate IC strategies and behaviours when managing HeV cases is a matter of personal health choice. Social scientists have developed various cognitive models to help predict health behaviours by examining a number of cognitive skills: *knowledge, motivation, readiness to change behaviour, expected outcomes, risk perception, perception of peer behaviour and beliefs about peer opinion of targeted behaviour* [48]. When HeV first emerged many of these variables would not have scored highly amongst private veterinarians. Veterinarians have been shown to make evidence-based IC decisions [49]. With HeV outbreaks occurring sporadically up until 2010 (13 self-limiting outbreaks with more than four years between the second and third outbreaks and the third and fourth outbreaks) [6], there were few opportunities to gain epidemiological and clinical knowledge about, or management experience of the disease. The full spectrum of clinical signs in horses, non-specific for the most part, was revealed over decades. As more cases accumulated it became obvious that infected horses could also excrete virus during the incubation period [50]. With such a high clinical variability, equine HeV could only be confirmed by laboratory tests, a process that was not deemed straightforward by all veterinarians [26]. Many participants felt that although they understood the principles of zoonotic risk mitigation, they generally lacked experience dealing with emerging zoonoses in equine practice. Thus, HeV-related risk perception, motivation and readiness for IC behavioural change was low during this time, even though during the first two outbreaks three people became infected, two fatally. Participants’ behaviours seemed to have also been influenced by: peer perceptions; beliefs about IC and zoonoses; and a strong professional identity based on the long standing belief that using PPE was a sign of weakness and that acquiring a zoonosis was an achievement not a reflection of malpractice [51]. These beliefs may have been stronger amongst older participants who had graduated longer ago as they are likely to have received less theoretical and practical biosecurity than those who graduate more recently [44].

**Figure 1:**
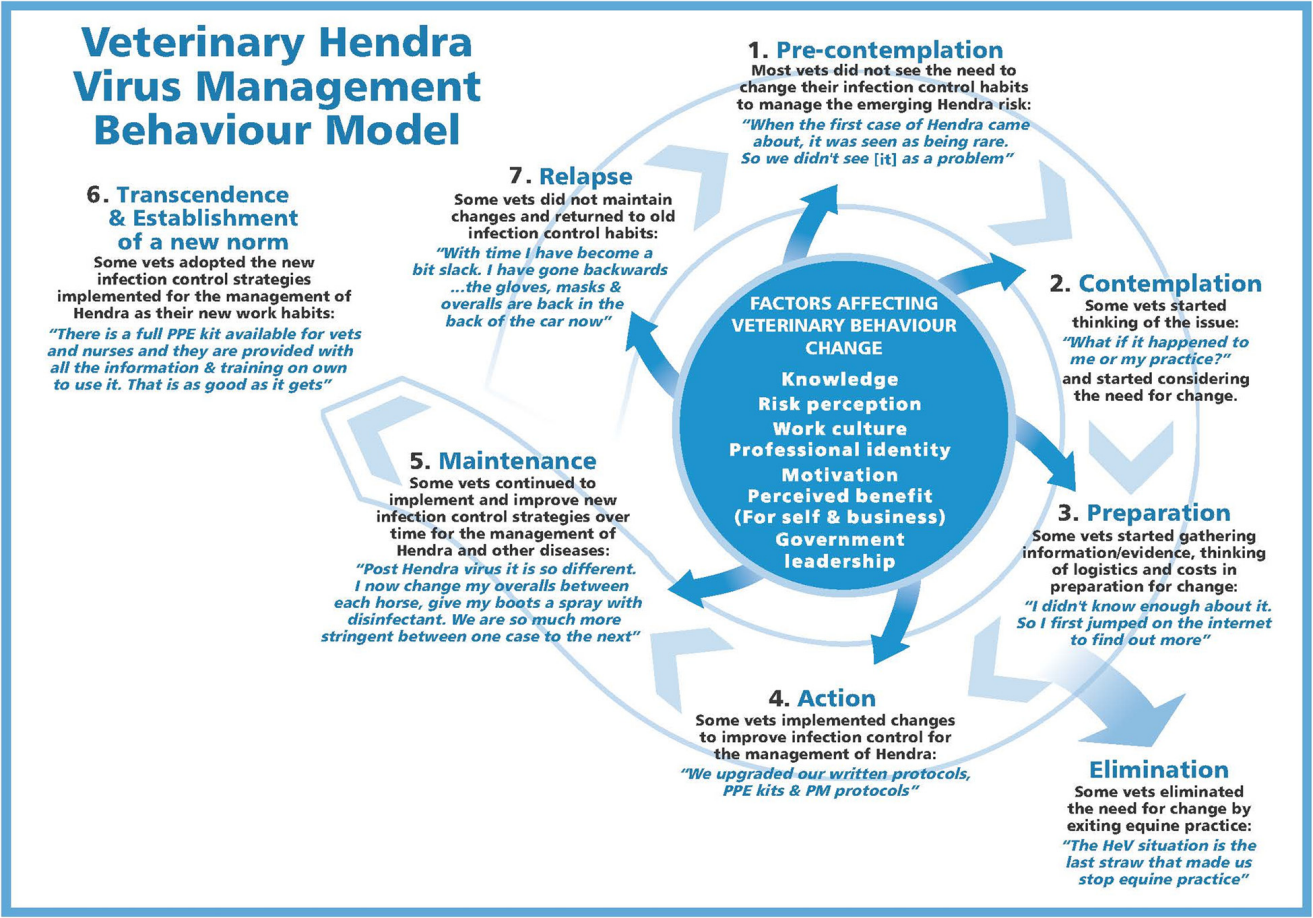
**Veterinary Hendra virus management behaviour model, adapted from trans-theoretical model of health behaviour change.** Model based on results of a qualitative study investigating barriers to infection control behaviour relevant to Hendra virus management in private veterinary practice in Queensland, Australia, conducted with 21 participants during 2009–2010 [52,53].

Veterinarians and government actions and decisions relating to the management of HeV followed the typical stages of behaviour change: *pre-contemplation* (behavioural change seen as not needed); *contemplation* (behavioural change under consideration); *preparation* (for behavioural change); *action* (behavioural change implementation); *maintenance* (of behavioural change); *transcendence* (behavioural change becomes the new behavioural norm); and sometimes relapse (reverting to old behaviours) [52,53]. The progression through these stages is determined by a number of cognitive variables (Figure 1). In the context of the emergence of HeV, there was at first disbelief that HeV would recur or spread to other areas, and as the government did not recommend any particular changes, veterinary IC behaviours remained unchanged. With recurring HeV outbreaks and additional human infections, there was a slow recognition that changes to veterinary IC were necessary. Some IC changes were made by some veterinarians but these were not always sustained as the required financial and logistic investment could not be justified long-term because of the low frequency of HeV outbreak and the lack of government commitment to a clear HeV management plan. The deaths of two veterinarians in 2008 and 2009 triggered government into finalising their HeV management plan, formulate and widely distribute clear recommendations to private veterinarians and provide more training and field support to equine veterinarians through their emergency response unit. In 2010 all private veterinarians registered in Queensland, received a comprehensive information package from the government. The last three participants to be interviewed for this study had received this package prior to their interview and they were mostly satisfied with this information. Since then there have been no further human infections with HeV even though, in the winter of 2011, there was a marked increase in the number of HeV outbreaks in Queensland and northern New South Wales. Thus, the changes made by the government and the private veterinarians appear to have lowered the risk of human exposure and therefore infection risks for veterinarians and the wider population.

## Conclusion

Unlike other zoonoses, which emerged rapidly in human populations causing epidemics (e.g., severe acute respiratory syndrome (SARS), swine influenza (H1N1) in 2009), HeV was slow to emerge, the index cases occurred in horses rather than in humans and HeV did not affect public health on a grand scale. Unlike SARS and swine influenza, HeV is a low-incidence high-consequence pathogen [54]. This type of slow emerging zoonosis, which only affects human health occasionally, comes with its own set of unique challenges. Because they recur infrequently, remain geographically localised or have a limited effect on public health, the health risk they pose to humans may be difficult to assess. However, there is a need to recognise the potential significance of this type of zoonosis earlier and implement risk mitigation measures accordingly. Failing to do so, may put people at risk, in particular professionals such as farmers, zoo and wildlife carers, and veterinarians who may come in contact with an infected animal. However, it would be unrealistic to mobilise the same level of resources used for the management of zoonoses such as SARS or swine influenza. Nevertheless, it may be possible to develop a template framework for the management of slowly emerging zoonoses, which could include: improving veterinary IC and emerging zoonoses management preparedness through education; better communication between government authorities, veterinarians and the public; better recognition of the biosecurity and public health role and services provided by private veterinarians; consultation with private veterinarians when developing zoonoses management plans compatible with running private practices; a clearer definition of the respective roles of government, professional agencies and private veterinarians using officially ratified agreements; and better and equitable government support for the management of emerging zoonoses to all private veterinarians across all affected geographic areas. A closer collaboration and mutual understanding between private veterinarians and government could be the key to improving adaptability of both parties to slowly emerging or infrequent zoonoses.

## Abbreviations

HeV: Hendra virus
IC: Infection control
PPE: Personal protective equipment
AVA: Australian veterinary association
EVA: Equine veterinarians Australia
WHS: Workplace health and safety
SARS: Sever acute respiratory syndrome
